# A systematic review of oncosurgical and quality of life outcomes following pelvic exenteration for locally advanced and recurrent rectal cancer

**DOI:** 10.1308/rcsann.2023.0031

**Published:** 2024-02-16

**Authors:** J Maudsley, RE Clifford, O Aziz, PA Sutton

**Affiliations:** ^1^ Colorectal and Peritoneal Oncology Centre, Christie NHS Foundation Trust, UK; ^2^ Division of Cancer Sciences, University of Manchester, UK; ^3^ Institute of Translational Medicine, University of Liverpool, UK

**Keywords:** Locally advanced rectal cancer, Locally recurrent rectal cancer, Quality of life, Outcomes

## Abstract

**Introduction:**

Pelvic exenteration (PE) is now the standard of care for locally advanced (LARC) and locally recurrent (LRRC) rectal cancer. Reports of the significant short-term morbidity and survival advantage conferred by R0 resection are well established. However, longer-term outcomes are rarely addressed. This systematic review focuses on long-term oncosurgical and quality of life (QoL) outcomes following PE for rectal cancer.

**Methods:**

A systematic review of the PubMed^®^, Cochrane Library, MEDLINE^®^ and Embase^®^ databases was conducted, in accordance with the PRISMA (Preferred Reporting Items for Systematic reviews and Meta-Analyses) guidelines. Studies were included if they reported long-term outcomes following PE for LARC or LRRC. Studies with fewer than 20 patients were excluded.

**Findings:**

A total of 25 papers reported outcomes for 5,489 patients. Of these, 4,744 underwent PE for LARC (57.5%) or LRRC (42.5%). R0 resection rates ranged from 23.2% to 98.4% and from 14.9% to 77.8% respectively. The overall morbidity rates were 17.8–87.0%. The median survival ranged from 12.5 to 140.0 months. None of these studies reported functional outcomes and only four studies reported QoL outcomes. Numerous different metrics and timepoints were utilised, with QoL scores frequently returning to baseline by 12 months.

**Conclusions:**

This review demonstrates that PE is safe, with a good prospect of R0 resection and acceptable mortality rates in selected patients. Morbidity rates remain high, highlighting the importance of shared decision making with patients. Longer-term oncological outcomes as well as QoL and functional outcomes need to be addressed in future studies. Development of a core outcomes set would facilitate better reporting in this complex and challenging patient group.

## Introduction

There are approximately 43,000 new cases of colorectal cancer diagnosed in the UK each year, with over a quarter of those being rectal in origin.^1^ Worldwide, rectal cancer is the eighth most common cancer subtype with 732,210 new cases diagnosed each year^2^ and mortality is expected to rise by 60% ahead of 2035.^3^ Tumours that have breached the mesorectal fascia (T4 in the TNM [tumour, lymph nodes, metastasis] classification)^4^ are deemed to be locally advanced^5^ and account for up to 50–64% of annual cases in the UK.^6^ There is, however, international variation, with T4 tumours accounting for only 9% of cases in the Netherlands.^7^ The causal factors for this variation is unclear. Multidisciplinary teams will consider many factors when assessing the best oncological approach for each individual patient based on tumour anatomy, staging, evidence of nodal and metastatic disease, and patient comorbidities.^8^

Earlier stages of rectal cancer without evidence of metastasis or invasion can be treated successfully with surgery alone, with or without neoadjuvant chemoradiotherapy.^8^ A standard surgical approach to rectal cancer utilises the mesorectal fascia as a surgical excision plane in order to achieve clear oncological resection margins.^9,10^ For many locally advanced and recurrent rectal cancers, total mesorectal excision surgery is inadequate and a more extensive resection may be required.^6^ Frequently, this requires *en bloc* resection of the rectum, bladder and reproductive organs, with or without adjacent neurovascular structures and attached bone.^11^ Total PE involves the complete resection of all of these organs whereas partial PE has been described as the resection of two or more pelvic organs along with the tumour (with or without attached bone).^12–14^

Credited to enhanced surgical techniques and enriched perioperative care, PE outcomes have improved significantly over recent years.^15^ Data suggest that approximately 80% of patients with locally advanced rectal cancer (LARC) and 60% of those with locally recurrent rectal cancer (LRRC) will achieve a resection with microscopically clear (R0) margins.^16^ The 30-day mortality rate has decreased to less than 5%, with overall 5-year survival rates reported up to 70%.^17,18^ Owing to the extensive nature of this surgery, postoperative morbidity is high and can reach 80%.^19^

There is, however, a paucity of data on the impact of this surgery on long-term quality of life (QoL), including functional and psychological outcomes, which may help guide patient selection and decision making.^20^ Additionally, existing data regarding long-term survival following PE present significant heterogeneity, often reliant on subgroup analyses of different pelvic malignancies^21,22^ or not distinguishing between primary and recurrent rectal cancer.^23^ The aim of this systematic review was to appraise the current literature focusing on long-term outcomes following PE for LARC and LRRC in order to further understand the available evidence to facilitate patient selection and guide decision making for these complex procedures.

## Methods

A systematic literature review was performed and reported according to the PRISMA (Preferred Reporting Items for Systematic reviews and Meta-Analyses) guidelines.^24^ The protocol was registered in the PROSPERO database (CRD42022293491) prior to commencement.

A literature search for published full-text articles was undertaken by the investigators in January 2022 using the PubMed^®^, Cochrane Library, MEDLINE^®^ and Embase^®^ databases, and the search criteria string “(outcomes OR PROMS OR quality of life OR oncosurgical OR survival OR functional OR recurrence OR local recurrence OR distal recurrence) AND (pelvic exenteration surgery OR exenteration surgery) AND (locally advanced rectal cancer OR T4 rectal cancer OR recurrent rectal cancer OR locally recurrent rectal cancer)”. The search was restricted to the English language. However, no date restrictions were applied. Additional papers were sought by manually searching the references of relevant papers. Prior to screening, a search was performed to exclude duplicated results and duplicated datasets. Studies introducing and describing operative techniques alone were not included in the review.

Search results were initially included owing to a relevant title or abstract as screened by two reviewers (JM and PS). Discrepancy on inclusion was resolved through discussion and those papers were then read through in full. Randomised controlled trials, prospective cohort studies, case controlled studies, retrospective cohort studies and case series including adult patients (>18 years) were included. Studies were excluded after review according to the following criteria: containing <20 patients; not reporting standalone data for patients with LARC and/or LRRC; not reporting standalone data for patients undergoing PE as defined above; and not including long-term (>12 months) outcomes. All included papers therefore contained explicit data on patients with rectal cancer undergoing PE, and avoided those where the data were combined with other malignancies and procedure types.

Once eligible papers were identified and a final list of papers had been established, basic demographics as well as short-term and long-term (>12 months) data were extracted manually from each included study. Data extraction was performed by one author (JM) and verified by one other author (PAS). Microsoft Excel^®^ was used to tabulate and prepare the data for presentation and descriptive statistical analysis. Risk of bias was assessed independently by two reviewers (JM and PAS) using the MINORS (methodological index for non-randomised studies) tool.^25^ Owing to the nature of the data extracted, no meta-analysis was performed.

## Results

**Figure 1:**
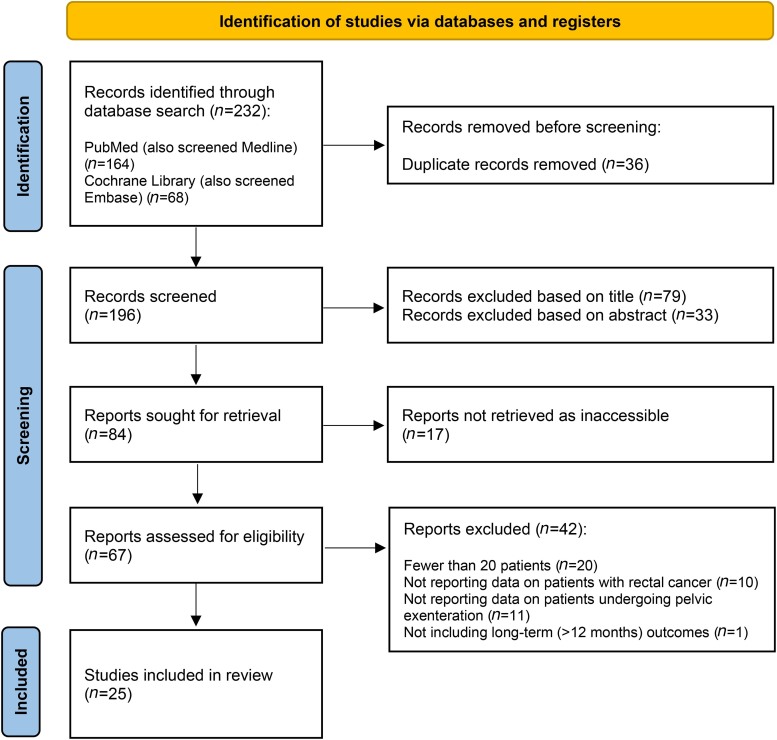
Flowchart of study selection

After removal of duplicates, 196 articles were screened and 84 were sought for full-text retrieval. Following application of the exclusion criteria, a total of 25 papers were included in this review, reporting on cohorts ranging from 22 to 2,472 patients. Figure 1 illustrates the study selection process.

### Study characteristics and baseline demographics

Of the 25 papers included in this review, none were randomised controlled trials, 8 were prospective cohort studies^12,26–32^ and 17 were retrospective studies.^15,33–48^ The risk of bias assessment is presented in Table 1, highlighting that all studies have a high risk of bias.

**Table 1:** Risk of bias assessment using the MINORS (methodological index for non-randomised studies) tool^25^ for the 25 papers included in the review. The scores were determined as follows: 0 = not reported; 1 = reported but inadequate; 2 = reported and adequate

**Study**	**Clearly stated aim**	**Inclusion of consecutive patients**	**Prospective data collection**	**Endpoints appropriate to study aim**	**Unbiased assessment of study endpoint**	**Follow-up period appropriate to study aim**	**<5% lost to follow-up**	**Prospective calculation of study size**	**Adequate control group**	**Contemporary groups**	**Baseline equivalence of groups**	**Adequate statistical analyses**	**Total**	**Risk of bias**
Alahmadi, 2021^12^	2	2	2	2	2	2	1	2	2	2	1	2	22/24	High
Balbay, 1999^33^	2	2	0	2	0	2	0	2	2	2	2	2	18/24	High
Bannura, 2006^34^	2	2	0	2	0	1	0	2	2	2	2	2	17/24	High
Choy, 2017^26^	2	2	2	2	0	2	1	1	2	2	2	1	19/24	High
Denost, 2020^27^	2	2	2	2	2	2	0	2	2	2	2	2	22/24	High
Domes, 2011^35^	2	2	0	2	0	2	1	1	N/A	N/A	N/A	N/A	14/16	High
Ferenschild, 2009^28^	1	2	0	2	0	2	2	2	0	2	0	2	15/24	High
Gannon, 2007^36^	2	2	0	2	0	2	1	0	N/A	N/A	N/A	N/A	9/16	High
Gawad, 2014^29^	2	2	0	0	0	2	0	2	N/A	N/A	N/A	N/A	8/16	High
Hagemans, 2018^37^	2	2	0	2	0	2	1	2	2	2	1	2	18/24	High
Hagemans, 2020^38^	2	2	2	2	0	2	1	2	0	2	2	2	19/24	High
Hsu, 2011^39^	2	1	0	2	0	2	1	0	N/A	N/A	N/A	N/A	8/16	High
Ishiguro, 2009^40^	2	2	0	2	0	2	1	2	N/A	N/A	N/A	N/A	11/16	High
Kakuda, 2003^41^	2	2	0	2	0	2	0	0	N/A	N/A	N/A	N/A	8/16	High
Kazi, 2021^42^	2	2	0	2	0	2	1	2	2	2	2	2	19/24	High
Kelly, 2019^15^	2	2	0	2	0	0	0	2	0	2	2	2	14/24	High
Kelly, 2020^43^	2	2	0	2	0	2	1	0	N/A	N/A	N/A	N/A	9/16	High
Meterissian, 1997^44^	2	2	0	2	0	2	2	2	N/A	N/A	N/A	N/A	12/16	High
Moriya, 2003^45^	2	1	0	2	0	2	2	2	2	2	0	2	17/24	High
Nielsen, 2012^30^	2	2	0	2	0	2	2	0	0	2	2	2	16/24	High
Pellino, 2018^46^	2	2	0	2	0	2	1	2	N/A	N/A	N/A	N/A	11/16	High
Radwan, 2015^31^	2	2	0	2	0	2	1	0	2	2	2	2	17/24	High
Radwan, 2015^47^	2	2	0	2	0	2	0	0	N/A	N/A	N/A	N/A	8/16	High
Rottoli, 2017^48^	2	2	0	1	0	2	0	2	0	2	2	2	15/24	High
Wiig, 2002^32^	2	2	0	1	0	2	1	0	0	2	2	2	14/24	High

The overall number of patients included in these studies was 5,489, of whom 4,744 were of interest. In total, 2,726 patients (57.5%) had LARC and 2,018 (42.5%) had LRRC, with the majority of studies (*n*=15) combining the two groups for outcomes. Seven studies focused on LARC alone,^31,34,40,43,45–47^ with three reporting LRRC cancer alone.^26,29,41^ The following exenteration types were reported: total PE (*n*=17, 68%), PE (*n*=7, 28%), posterior PE (*n*=6, 24%), anterior PE (*n*=3, 12%), extended PE (*n*=2, 8%), partial PE (*n*=1, 4%) and supralevator exenteration (*n*=1, 4%).

The median patient age across the included studies ranged from 45.0 to 72.5 years. Three papers did not disclose the sex ratio of study participants. However, among the 22 that did, the predominant sex was male (*n*=2,723, 57%). The median follow-up duration ranged from 14.5 to 68.0 months. Use of neoadjuvant treatment was reported in the majority of studies. Specifically, the use of chemoradiotherapy was reported in 12 studies (48%), chemotherapy in 8 (32%) and radiotherapy in 10 (40%). Adjuvant treatment was reported in nine studies only: chemoradiotherapy in two (8%), chemotherapy in five (20%) and radiotherapy in four (16%). Finally, eight studies (32%) reported the use of intraoperative radiotherapy. A summary of the included studies is given in Table 2.

**Table 2:** Study characteristics and patient demographics from the 25 papers included in the review

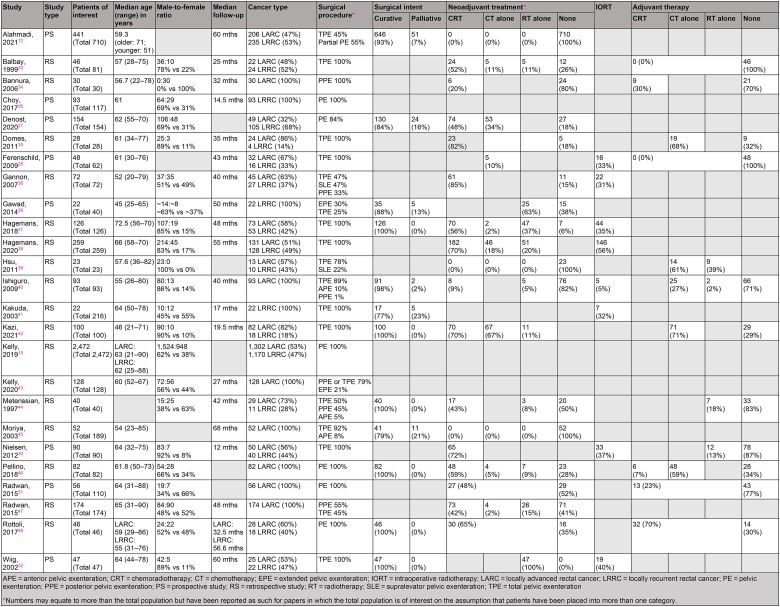

### Short-term outcomes

#### Surgical outcomes

Sixteen papers reported median operative time (210–600 minutes) and median blood loss (675–3,800ml). The median length of hospital stay ranged between 9.3 and 29.1 days, with the median length of stay in a higher level of care ranging from 2.0 to 3.3 days. Overall, there were 81 deaths within 30 days of surgery (1.7%), with 4 studies indicating a 30-day mortality rate of 0%. Conversely, one study reported a 90-day mortality rate of 8.7%^37^ and seven studies did not comment on 30-day mortality at all.

Two papers did not disclose overall postoperative complication rates,^35,44^ with only fifteen studies using the established Clavien–Dindo classification. In total, 2,170 patients (52.0%) were reported to have developed a postoperative complication, with wound infections (*n*=432, 19.9%) and gastrointestinal complications (*n*=345, 15.9%) being the most common. A total of 604 patients across 15 studies had to return to theatre (12.7%). Four studies reported an unplanned hospital readmission rate ranging between 14.1% and 45.5%.^29,38,41,43^ A summary of the short-term outcomes from included studies can be found in Table 3.

**Table 3:** Summary of short-term outcomes from the 25 papers included in the review

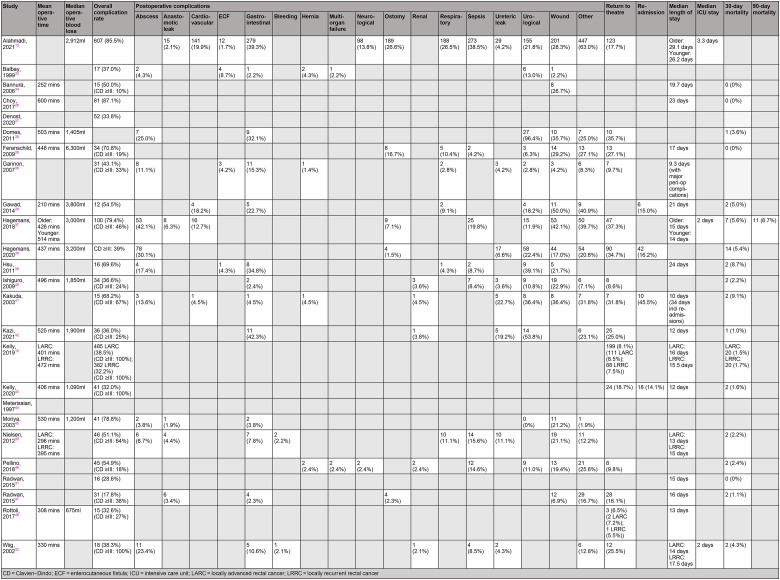

#### Oncological outcomes

TNM classification of tumours was reported sporadically. Overall R0 resection rates ranged between 57.4% and 100%, with one study noting a 100% R0 resection rate (*n*=40).^44^ R0 rates specific to LARC and LRRC cohorts ranged from 23.2% to 98.4% and from 14.9% to 77.8% respectively. Five papers gave R0 resection rates for LARC alone while two studies reported R0 rates for LRRC alone. Recurrence rates following PE ranged from 3.2% to 68%. Three studies mentioned unspecified recurrence rates of 13–33%, with the remaining providing individual recurrence rates specific to local recurrence (4.3–68%), distant recurrence (11–46%) and both (3.2–61%). Two studies reported median disease-free intervals of 11 and 20 months.^39,41^ Table 4 summarises the oncological outcomes following PE for LARC and LRRC.

**Table 4:** Summary of oncological and survival outcomes following pelvic exenteration for LARC and LRRC

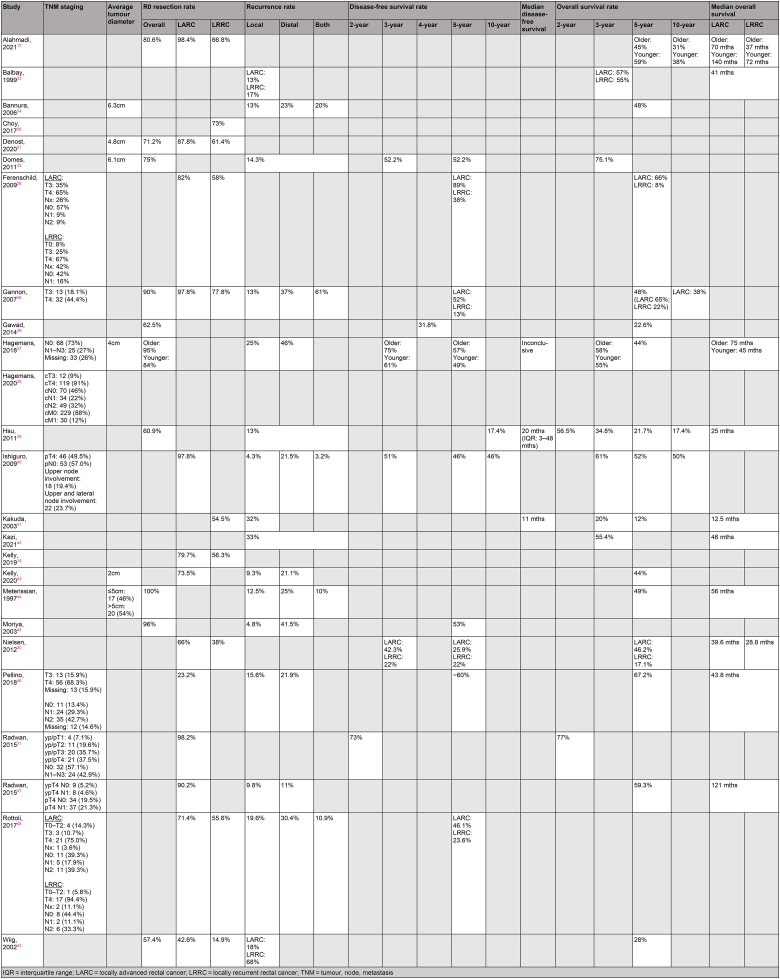

### Long-term outcomes

#### Survival

Eleven studies (44%) reported disease-free survival, the majority giving these as five-year rates ranging from 13% to 89%. Where reported, two, three, four and ten-year disease-free survival rates were 73%, 22–75%, 31.8% and 17.4–46% respectively. The reported median overall survival ranged from 12.5 to 140 months. Overall five-year survival rates were only noted in 15 studies (60%); these ranged from 8% to 67.2%. Where reported, two, three and ten-year overall survival rates ranged from 56.5% to 77%, 20% to 75.1% and 17.4% to 50% respectively.

#### Quality of life and functional outcomes

Only four of the studies included in this review investigated QoL outcomes, with considerable variation in the tools used and timepoints studied.^12,26,27,31^ QoL scores were most commonly recorded at baseline and at 12 months following surgery. Overall, patient scores reflected little change in QoL during this period of time.

The majority of the QoL metrics returned to baseline (or were only slightly lower) at 12 months. Baseline scores were, however, generally low, with a QLQ-C30 symptom score of 10.3/100,^31^ and SF-36^®^ scores of 40.7–46.4/100 for older patients and 42.0–43.3/100 for younger patients.^12^

With respect to the SF-36^®^ questionnaire, the mental component scores improved at 24 months compared with baseline for both the older and younger patient subgroups (52.3 vs 46.4 and 48.4 vs 43.3 respectively).^12^ Similarly, the physical component scores for older patients improved at 24 months (44.7 vs 40.7). Conversely, these values deteriorated slightly for younger patients (41.2 vs 42.0), who may have had greater initial physical ability and could therefore have experienced a greater decline following surgery. The FACT-C values show trends in improvement by 60 months compared with baseline for both patient groups: 98.8 vs 94.9 for older patients and 98.6 vs 9.0 for younger patients.^12^

The QoL outcomes from each included study are summarised in Table 5. None of the studies reported further functional outcomes following PE surgery for either LARC or LRRC cohorts.

**Table 5:** Summary of the quality of life data reported by four of the included studies

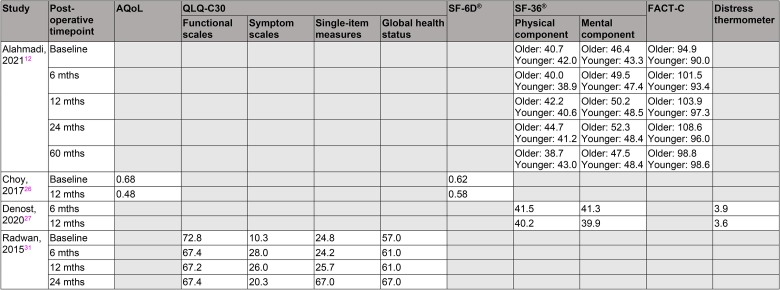

## Discussion

PE has an established and significant impact on patients with advanced pelvic malignancy, both physically and psychologically. The current literature contains extensive reports of the short-term and oncological outcomes following surgery to aid personalised clinician and patient shared decision making although data on long-term outcomes and QoL still remain sparse.

Our review focused on patients with LARC and LRRC, including comparatively large studies where long-term outcomes had been explicitly reported for the groups of interest. Across the cohort, morbidity was found to be high, ranging from 17.8% to 87.1%, with half of the included studies having a rate of over 50%. Overall 30-day mortality rates ranged between 0% and 9.1%. Previous studies have noted 30-day mortality rates of 0–25%, demonstrating that with advances in technique and perioperative management, patients are undergoing surgery of this nature relatively safely.^6^ Overall R0 resection rates were between 57.4% and 100%, with rates of 14.9–77.8% reported for those with LRRC. Higher R0 rates were seen in patients undergoing multivisceral resection.^49^

Long-term data with respect to recurrence and survival are less abundant for this patient cohort. Disease-free survival as most frequently reported at three and five years after surgery (22–75% and 13–89% respectively), with only a handful of studies reporting beyond the five-year timepoint. Comparatively, five-year local recurrence rates for patients with low rectal cancer undergoing abdominoperineal excision or coloanal anastomosis for earlier tumours have been noted as 7.9% and 5.3% respectively.^50^ Analysis of five-year overall survival revealed considerable variation (12–67.2%), likely reflecting different patient populations with variation in complication, resection and recurrence rates. Such wide variation in figures presumably represents the considerable heterogeneity in this patient population, making it difficult to draw firm conclusions.

Reports of functional and societal outcomes beyond the use of validated QoL metrics were absent across all included studies. These may include concepts such as returning to employment, social activities and family responsibilities, as have been reported in other studies comparing laparoscopic with other surgical techniques for colorectal cancer.^51,52^ The four studies that formally reported QoL outcomes used a combination of six questionnaires and surveys: AQoL, QLQ-C30, SF-6D^®^, SF-36^®^, FACT-C and the distress thermometer.^12,26,27,31^

It is important to acknowledge the evidently low QoL scores at baseline and at 12 months after surgery. These data provide hindsight that the initial 12 months following PE will demonstrate the poorest QoL, highlighting the need for early mitigation (even prior to surgery) in order to optimise QoL outcomes.

Data from a meta-analysis suggest that patients who receive prehabilitation prior to cancer surgery have an accelerated recovery time.^53,54^ Identifying high risk patients prior to surgery can ensure additional prehabilitation and preoperative assessment on an individual patient basis.^55^ Malnutrition is also frequently reported in patients with cancer undergoing complex major surgery.^53^ Incorporating an assessment of malnutrition and nutritional optimisation into a prehabilitation programme has been associated with improved perioperative outcomes and reduced hospital stay for patients with locally advanced oesophageal cancer.^56^ Similar improvements may be seen following implementation in the cohort of patients with LARC, which may improve postoperative physiological and psychological states, and ultimately QoL.

While trends of improved QoL beyond the 12-month timepoint were unexpected, this could be explained by patients becoming more familiar with and “used to” their new way of life. Although these data may indicate a timely recovery period, this could also help reassure and inform patients that despite the invasiveness of this surgical approach, the data do support improvements in QoL even beyond the 12-month timepoint after surgery. Several of the QoL instruments used demonstrate these positive trends up to 24 and 60 months following surgery. Combined with improved R0 resection rates and acceptable mortality rates, these findings continue to justify this radical treatment approach for these patients.

Such data could inform a shared decision making approach with patients, ensuring their values and preferences are considered in the decision making process. Efforts to improve patient engagement are vital considering the extensive impact that PE has on patients’ wellbeing and lifestyle. Patient decision aids are a valuable clinical tool that complements this approach, containing information regarding the advantages and disadvantages of the clinical options available, thereby allowing patients to determine which would be of greater concern to them personally.^57^ Despite evidence supporting the effective clinical use of patient decision aids, such tools have not yet been validated for patients with LARC or LRRC.^58,59^ However, Williams *et al* provide promising foundations for overcoming this following the design and evaluation of such aids for this patient group.^60^

The tools used in these studies are all validated means of assessing QoL in patients with cancer. With the exception of the distress thermometer, each questionnaire considers several factors to formulate an overall score assessing multiple aspects of the patient’s QoL, including symptom related questions, and psychological and physical factors.^61–63^ For example, the AQoL, SF-6D^®^ and SF-36^®^ questionnaires explore independent living, and psychological and physical wellbeing as well as social relationships. Nevertheless, utilising overall scores does not highlight specific areas that may be affected more than others following PE as deterioration in certain domains may be masked by improvements in others.

While the FACT-C instrument is a tool recommended specifically for patients with colorectal cancer, it is not validated for recurrent cancer and therefore risks misinterpretation.^64^ The dedicated LRRC-QoL patient reported outcome measure currently under development and validation by Harji *et al* will serve as a useful tool in the prospective study of health related QoL after surgery for LRRC.^65^

This study has deliberately focused on patients undergoing PE as defined a priori, for rectal cancer, and has only included studies containing a comparatively larger sample (of ≥20 patients) giving independent data for the cohorts of interest. Owing to low numbers of patients, many studies combine either procedures or tumour types for reporting. Despite the strict criteria applied in this review, there remains considerable heterogeneity in the reported outcomes, which makes any firm conclusions from this study difficult. This could possibly be improved by development of a core outcomes set following the guidelines of the COMET (Core Outcome Measures in Effectiveness Trials) initiative.^66^

Other methodological limitations include the retrospective nature of most of the included studies with a small sample size. All studies were at high risk of bias and consequently, confounding factors cannot be accounted for. Clinicians must continue to treat and counsel patients using the available evidence, for which we have attempted to give a clinically useful overview.

## Conclusions

This review of current evidence demonstrates that PE is safe, with a good prospect of R0 resection and acceptable mortality rates in selected patients. Morbidity rates remain high, highlighting the importance of shared decision making with patients around their treatment options. Nevertheless, this review also highlights that there is significant heterogeneity in the cohorts studied and wide variation in outcomes reported. In particular, longer-term oncological outcomes as well as QoL and functional outcomes need to be addressed in the design of future studies. Development of a core outcomes set would facilitate better reporting in this complex and challenging patient group.
